# 2D layered SiP as anisotropic nonlinear optical material

**DOI:** 10.1038/s41598-021-85938-4

**Published:** 2021-03-18

**Authors:** Huseyin Sar, Jie Gao, Xiaodong Yang

**Affiliations:** Department of Mechanical and Aerospace Engineering, Missouri University of Science and Technology, Rolla, MO 65409 USA

**Keywords:** Nonlinear optics, Two-dimensional materials

## Abstract

Two-dimensional (2D) material of silicon phosphide (SiP) has recently been shown as a promising optical material with large band gap, fast photoresponse and strong anisotropy. However, the nonlinear optical properties of 2D SiP have not been investigated yet. Here, the thickness-dependent in-plane anisotropic third-harmonic generation (THG) from the mechanically exfoliated 2D layered SiP flakes is reported. The crystal orientation of the SiP flake is determined by the angle-resolved polarized Raman spectroscopy. The angular dependence of the THG emission with respect to the incident linear polarization is found to be strongly anisotropic with the two-fold polarization dependence pattern. Furthermore, the effect of the SiP flake thickness on the THG power is analyzed.

## Introduction

The era of 2D layered materials, also known as van der Waals materials, has begun by the exfoliation of bulk graphite into monolayer graphene with extraordinary properties^1^. Beyond graphene, 2D materials such as transition metal dichalcogenides (TMDs) like MoS_2_ and WSe_2_, halides (MX_2_ with X being Cl, Br, I^2^) and carbides/nitrides (MXenes^3–5^) have gained more attraction due to their superior electrical, optical, mechanical, thermoelectric, energy storage and sensing properties. Owing strong light-material interaction with broad spectral coverage range and tunable band gap, 2D materials are endorsed as promising optical materials. Besides to the linear optical properties, 2D materials are becoming prominent as promising nonlinear optical materials because of the high power conversion efficiency and large nonlinear optical susceptibility. Therefore, 2D materials have been accounted on several nonlinear optical applications including third-harmonic generation^6,7^, second-harmonic generation (SHG), self-phase modulation^8,9^, nonlinear Kerr effect^10,11^, and four-wave mixing (FWM)^12^. Furthermore, 2D materials with low crystal symmetry such as BP^13^, GeSe^14^, GeAs^15^, ReS_2_^16^ and SnSe_2_^17^ provide the opportunities for controlling the anisotropic physical properties with respect to the in-plane crystal orientation, giving a new degree of freedom for the angle-dependent manipulation. The anisotropic optical response empowers many novel photonic devices such as polarization sensors^18^, synaptic devices^19^, polarization sensitive photodetectors^20,21^, and directional memories^22^. The integration of these anisotropic 2D materials with strong nonlinear optical responses into the photonic devices is important in the development of future nanoscale on-chip optical circuits^23^ and quantum photonics^24^.

In this manner, SiAs, GeAs, GeAs_2_, GeAs, GeP and SiP as the members of a novel group IV-V 2D semiconductor family have gained a growing attention^25–27^ due to their promising linear optical properties and low crystal symmetries. Specifically, SiP has been shown as an excellent optical material with a large band gap of 1.71 eV, fast photoresponse and strong anisotropy^28,29^. However, the intrinsic nonlinear optical properties of 2D layered SiP have not been investigated yet. In this study, the strong in-plane anisotropic THG in the exfoliated SiP flakes with different thicknesses is investigated. The angle-resolved polarized Raman spectra and the anisotropic THG responses of SiP thin flakes are measured and analyzed systematically. The effect of the SiP flake thickness on the THG power is also investigated experimentally and the extinction coefficient of SiP at the THG wavelength of 520 nm is estimated by theoretical analysis.

## Results

### Determination of SiP crystal orientation with Raman spectroscopy

**Figure 1:**
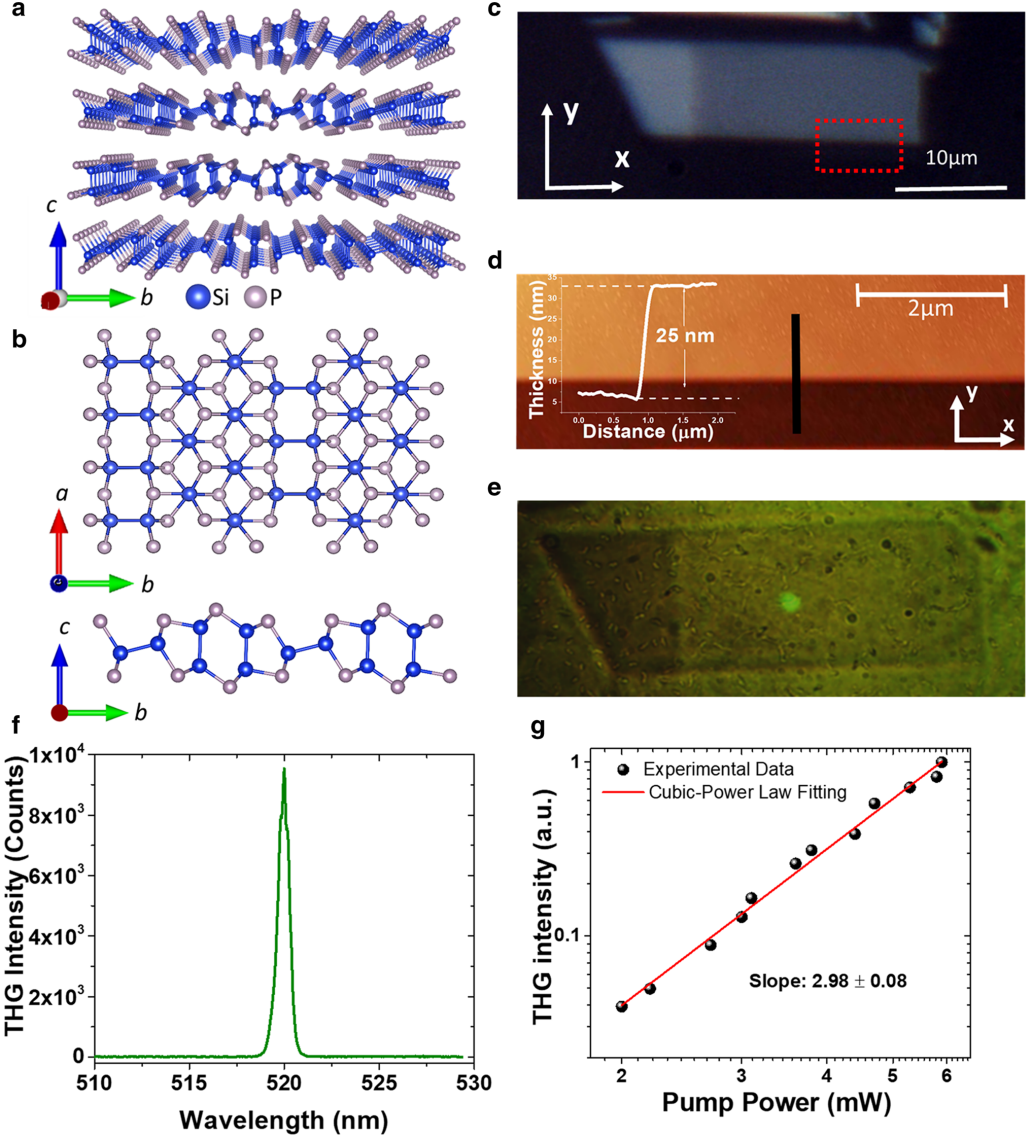
**(a)** The perspective view schematic of the crystal structure of 2D SiP. **(b)** The top view and side view schematics of a SiP monolayer. **(c)** The optical reflection microscope image of one exfoliated SiP flake with the laboratory axes *x* and *y*. Scale bar is 10 μm. The red dashed rectangle represents the region that the AFM is measured. **(d)** The AFM image of the SiP flake with the height profile through the black line as the inset figure. Scale bar is 2 μm. **(e)** The optical transmission microscope image of the flake with the green THG emission spot. **(f)** The spectrum of THG emission with the emission wavelength of 520 nm by using the 1560 nm pulsed laser excitation. **(g)** The measured THG emission intensity as a function of the incident pump power (black spheres) with the cubic-power law fitting curve (solid red line).

The in-plane anisotropic optical properties of SiP are a result of its low lattice symmetry. As a member of the group IV-V 2D semiconductor family, SiP exhibits the orthorhombic lattice with Cmc2_1_ space group. The individual monolayers of SiP are held together by weak van der Waals interactions along the *c*-axis with an *AB* stacked layers to form the multilayer structure^30^. The perspective view of SiP crystal lattice is depicted in Fig. 1a, while the side view and top view of a SiP monolayer are depicted in Fig. 1b. The armchair and zigzag edge terminations along *b*-axis and *a*-axis are also illustrated in Fig. 1b.

Figure 1c shows the optical reflection microscope image of a mechanically exfoliated SiP flake with the laboratory frame *x-* and *y-*axes. The angle-resolved polarized Raman spectroscopy measurements are used to determine the armchair direction (*b*-axis) and zigzag direction (*a*-axis), where *x-* and *y*-axes are assigned as the armchair and zigzag directions accordingly. The thickness and surface characterization of the exfoliated SiP flakes are evaluated by the atomic force microscopy (AFM) measurements, as shown in Fig. 1d. The height profile extracted from the black line on the AFM image is shown as the inset of Fig. 1d, and the flake thickness is 25 nm. Figure 1e is the optical transmission microscope image of the flake, with the green THG emission spot excited by a 1560 nm pulsed pump laser with the spot size of 2.5 µm. The typical THG emission spectrum is shown in Fig. 1f where the emission wavelength is 520 nm. The cubic power-law dependence of the THG emission as a function of the pump power plotted in Fig. 1g is another proof of the THG process. The fitting is well achieved with a slope of $2.98\pm 0.08$, confirming the occurrence of THG emission.

**Figure 2:**
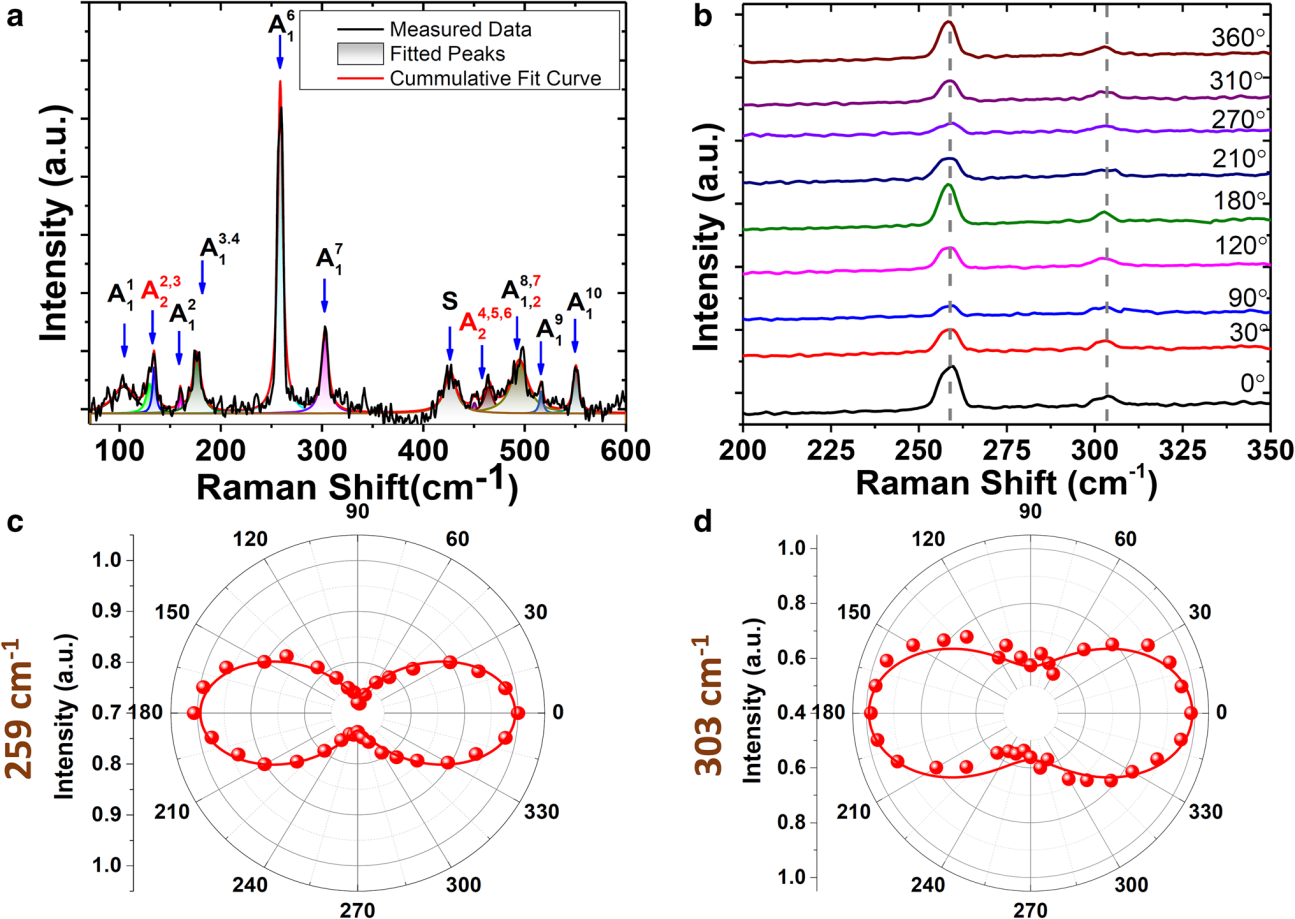
**(a)** The total Raman spectrum measured from the exfoliated 25 nm-thick SiP flake. **(b)** The angle-resolved polarized Raman spectra of the SiP flake acquired in the parallel configuration for different pump polarization angles. **(c)**, **(d)** the polar plots of the Raman intensities of ${\mathrm{A}}_{1}^{6} (259 {\mathrm{cm}}^{-1})$ and ${\mathrm{A}}_{1}^{7} (303 {\mathrm{cm}}^{-1})$. The red spheres are the experimental data and the solid curves correspond to the data fitting by Eq. (2).

In Fig. 2a, the measured total Raman spectrum from the 25 nm-thick SiP flake is plotted, where the analyzer after the sample is removed. Since the measurements are carried out on a thin flake with the thickness of 25 nm, the Raman intensities of some modes are weak. In order to extract the exact Raman mode shifts and intensity values, each Raman mode has been fitted by a Gaussian function as shown in Fig. 2a. The Raman active modes of 2D layered SiP are along different atomic displacement directions in the *ab*-plane. Here, the estimated peak centers of Raman modes belonging to the vibrational direction along *b-*axis are $104.3, 176.42, 258.72, 302.66, 494.41, 516.46,$ and $550.71 {cm}^{-1}$ which are assigned as ${A}_{1}^{1}$ to ${A}_{1}^{10}$ modes, while the estimated peak centers of Raman modes belonging to the atomic displacement direction along *a*-axis are $129.54, 134.22, 426.12, 450.32,$ and $464.43 {cm}^{-1}$ which are assigned as ${A}_{2}^{1}$ to ${A}_{2}^{7}$ modes. At this point,${A}_{1}^{\mathrm{3,4}},$ ${A}_{2}^{\mathrm{2,3}}$,${A}_{2}^{\mathrm{4,5},6}$ and ${A}_{\mathrm{1,2}}^{\mathrm{8,7}}$ modes refer to the overlapped Raman modes due to the adjacent vibration frequencies and weak intensities. These observed Raman modes match well with the previously reported Raman spectrum of SiP^28,31^. The Raman spectrum is not just utilized as the material defining tool but also used to determine the crystal orientation by resolving the polarization dependence behavior of the Raman modes. In this manner, the angle-resolved polarized Raman spectroscopy is commonly used to determine the crystal orientation^15,32–35^. Here, the parallel configuration in the angle-resolved polarized Raman spectroscopy is utilized where the analyzer direction is set to be parallel with the excitation polarization direction. The SiP flake sample is fixed with the assigned *x* and *y* laboratory axes as depicted in Fig. 1c. The laser beam polarization is varied by rotating a half-wave plate to sweep the angle $\theta$ relative to *x*-axis from $0^\circ$ to $360^\circ$ with a step of $10^\circ$. In Fig. 2b, the Raman spectra of the 25 nm-thick SiP thin flake for different laser beam polarizations under the parallel configuration are plotted. The intensity variations of the Raman modes at ${{A}_{1}^{6}:259 cm}^{-1}$ and ${A}_{1}^{7}:303 {cm}^{-1}$ are shown to have a period of $180^\circ$ which is an indication of the crystalline orientation dependency. On the other hand, the Raman mode frequencies are almost unchanged with respect to the incident laser polarization.

To further quantitatively analyze the periodical intensity variation of the strongest two Raman modes and determine the crystal orientation, the Raman selection rule is used to derivate the mode intensity function in the parallel configuration. The generalized form of Raman intensity is stated as:

1 $$ I\left( j \right) = \left| {d_{i} \cdot \overline{\overline{R}} \left( j \right) \cdot d_{s}^{T} } \right| $$

where ${d}_{i}$ and ${d}_{s}$ refer to the unit polarization vectors of the incident and scattered light, $j$ refers to the mode number and $\overline{\overline{R}}$ is the Raman tensor^36^. The intensities of ${A}_{1}$ and ${A}_{2}$ modes in the parallel configuration are expressed as:

2 $${I}^{\parallel }\left({A}_{1}\right)\propto {\left({cos}^{2}\left(\theta \right)+\frac{{c}_{1}}{{c}_{2}}\cdot {sin}^{2}\left(\theta \right)\cdot \mathrm{cos}\left({\phi }_{{c}_{1}{c}_{2}}\right)\right)}^{2}+{sin}^{4}\left(\theta \right)\cdot {\mathrm{cos}}^{2}\left({\phi }_{{c}_{1}{c}_{2}}\right)$$

3 $${I}^{\parallel }\left({A}_{2}\right)\propto {\left|c\right|}^{2}\cdot {{sin}^{2}(\theta )\cdot cos}^{2}(\theta )$$

where ${c}_{1}$, ${c}_{2}$, and $c$ are the constants of the Raman tensor, $\theta$ the linear polarization angle with respect to the crystal axis, and ${\phi }_{{c}_{1}{c}_{2}}$ represents the phase difference between ${c}_{1}$ and ${c}_{2}$*.* The angle-resolved Raman intensity variations of the ${259 \mathrm{cm}}^{-1}$ and $303 {\mathrm{cm}}^{-1}$ modes are shown as the polar plots in Fig. 2c,d, respectively. The red dots represent the experimental data and the red solid lines refer to the calculated Raman intensities from Eq. (2). The anisotropic Raman intensity variation shows a two-lobe pattern aligned along the armchair direction. As a result of that, the armchair and zigzag directions are assigned as *x-* and *y-*axes, respectively.

### Anisotropic THG in exfoliated SiP flakes with different thicknesses

The previously studied anisotropic linear optical properties of SiP foreshadows strong anisotropic nonlinear optical responses, which can be revealed by employing the angle-resolved THG measurements for different flake thicknesses. The THG emission along the armchair direction (${E}_{x}^{3\omega }$) and the zigzag direction (${E}_{y}^{3\omega }$) are measured by fixing the analyzer at $0^\circ$ and $90^\circ$ degrees, respectively. To quantitively analyze the THG emission and estimate the components of the third-order nonlinear susceptibility tensor ${\chi }_{im}^{(3)}$, the analytic expression of the emitted THG signal is derived by using the input field expression (${\mathbf{E}}_{{\varvec{I}}}$) and the third-order nonlinear susceptibility tensor of the orthorhombic SiP crystal. The electric field intensity of the linearly polarized input pump beam with the fundamental frequency ($\omega$) can be defined as:

4 $${\mathbf{E}}_{{\varvec{I}}}=\left|\mathbf{E}\right|\widehat{r}$$

5 $$\widehat{r}=\widehat{x}\mathrm{cos}\,\theta +\widehat{y}\mathrm{sin}\,\theta $$

where $\theta$ is the polarization angle. The third-order nonlinear susceptibility tensor of SiP is expressed with the known crystal structure (orthorhombic) and space group (Cmc2_1_) as^37,38^:

6 $${\chi }_{im}^{(3)}=\left[\begin{array}{c}{\chi }_{11}\\ 0\\ 0\end{array}\begin{array}{c}0\\ {\chi }_{22}\\ 0\end{array}\begin{array}{c}0\\ 0\\ {\chi }_{33}\end{array}\begin{array}{c}0\\ {\chi }_{24}\\ 0\end{array}\begin{array}{c}0\\ 0\\ {\chi }_{35}\end{array}\begin{array}{c}{\chi }_{16}\\ 0\\ 0\end{array}\begin{array}{c}0\\ 0\\ {\chi }_{37}\end{array}\begin{array}{c}{ \chi }_{18}\\ 0\\ 0 \end{array}\begin{array}{c}0\\ {\chi }_{29}\\ 0\end{array}\begin{array}{c}0\\ 0\\ 0\end{array}\right]$$

where $i$ equals to 1, 2, and 3 refers to $x,y,z$ respectively, and $m$ shows the mutual relation of the three components as follows:

7 $$\begin{array}{c}jkl xxx\\ m 1\end{array} \begin{array}{c}yyy\\ 2\end{array} \begin{array}{c}zzz\\ 3\end{array} \begin{array}{c}yzz\\ 4\end{array} \begin{array}{c}yyz\\ 5\end{array} \begin{array}{c}xzz\\ 6\end{array} \begin{array}{c}xxz\\ 7\end{array} \begin{array}{c}xyy\\ 8\end{array} \begin{array}{c}xxy\\ 9\end{array} \begin{array}{c}xyz\\ 0\end{array}$$

As a result of *x–y* plane excitation, the components of ${\chi }_{im}^{(3)}$ with $z$ terms are set to be zero. The THG electric field components will have only four non-zero terms left which is the result of removing *z*-axis dependent components. The resultant THG electric field expression is:

8 $${E}^{3\omega }=\left[\begin{array}{c}{E}_{x}^{3\omega }\\ {E}_{y}^{3\omega }\\ {E}_{z}^{3\omega }\end{array}\right]\propto {\varepsilon }_{0}{E}^{3}\left[\begin{array}{c}\left({\chi }_{11}{\mathrm{cos}}^{3}\theta +3{\chi }_{18}\mathrm{cos}\theta {\mathrm{sin}}^{2}\theta \right)\\ \left({\chi }_{22}{\mathrm{sin}}^{3}\theta +3{\chi }_{29}\mathrm{sin}\theta {\mathrm{cos}}^{2}\theta \right)\\ 0\end{array}\right]$$

Then the intensity of the emitted THG signal is written as:

9 $${I}_{x}^{3\omega }\propto {{(E}_{x}^{3\omega })}^{2}\propto {\left({\chi }_{11}{\mathrm{cos}}^{3}\theta +3{\chi }_{18}\mathrm{cos}\theta {\mathrm{sin}}^{2}\theta \right)}^{2}$$

10 $${I}_{y}^{3\omega }\propto {{(E}_{y}^{3\omega })}^{2}\propto {\left({\chi }_{22}{\mathrm{sin}}^{3}\theta +3{\chi }_{29}\mathrm{sin}\theta {\mathrm{cos}}^{2}\theta \right)}^{2}$$

**Figure 3:**
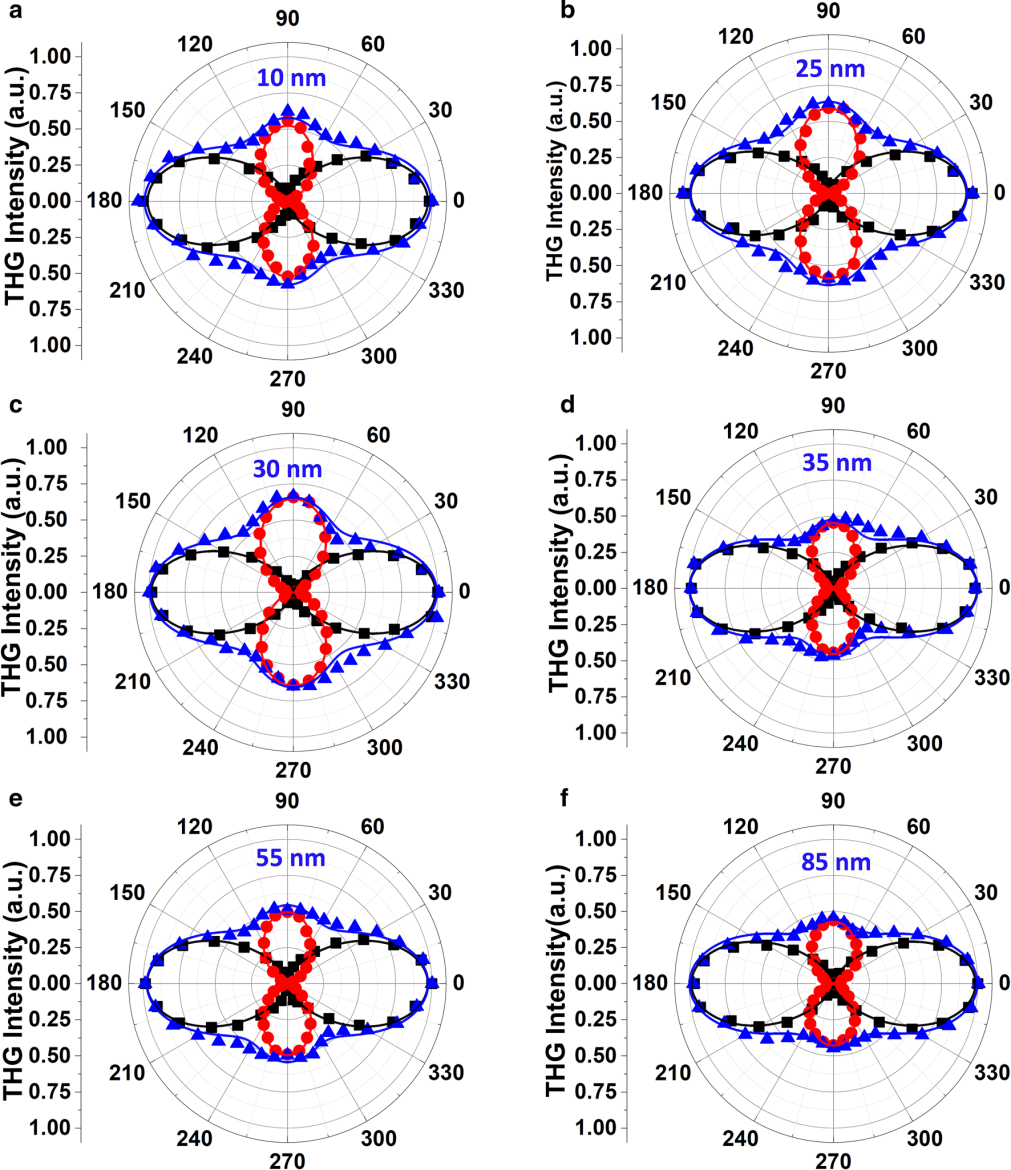
The angle-resolved THG intensity with respect to the incident linear polarization angle for the SiP flakes with thicknesses of **(a)** $10 \mathrm{nm}$, **(b)** $25 \mathrm{nm}$, **(c)** $30 \mathrm{nm}$, **(d)** $35 \mathrm{nm}$, **(e)** $55 \mathrm{nm}$ and **(f)** $85 \mathrm{nm}$. The black squares, red circles and blue triangles represent the measured *x*-component, *y*-component and total THG intensity, respectively. Solid black, red and blue curves refer to the data fittings by Eqs. (9) and (10).

In Fig. 3, the incident polarization angle-resolved THG emission plots of 2D SiP flakes with varying thicknesses from $10$ to $85 \mathrm{nm}$ are shown, in which the strong anisotropic nonlinear optical response of the 2D SiP is clearly observed. For all the polar plots, $0^\circ$ refers to the armchair direction and $90^\circ$ refers to the zigzag direction. The black squares and red circles are the measured THG emission components along the armchair direction (${I}_{x}^{3\omega }$) and the zigzag direction (${I}_{y}^{3\omega }$) respectively, while the blue triangles represent the total THG emission (${I}^{3\omega }$) with respect to the incident polarization angle. For all the flakes, the maximum THG signal is emitted as the input laser polarization is aligned along the armchair direction of the 2D SiP crystal, while the second maximum THG emission is occurred for the laser polarization along the zigzag direction of the crystal. The varying THG emission intensities for different incident polarization angles validate the strong anisotropic nonlinear optical response of 2D SiP. It is clearly shown that the input laser polarization angle where the maximum THG signal is obtained is in a perfect match with the angle of the armchair direction determined by the angle-resolved Raman measurements, manifesting that the nonlinear optical anisotropy of 2D SiP observed in THG measurements and the structural anisotropy determined by Raman measurements are in phase along the armchair and zigzag directions. Furthermore, the measured data is fitted by Eqs. (9) and (10) to estimate the components of the third-order nonlinear susceptibility tensor. The calculation results are depicted as the solid black, red and blue lines which refer to the ${I}_{x}^{3\omega }$, ${I}_{y}^{3\omega }$ and ${I}^{3\omega }$ in Fig. 3. The calculated data matches well with the measured data. The exfoliated SiP flakes with thicknesses of $10, 25, 30, 35, 55$ and $85 \mathrm{nm}$ exhibits very similar anisotropy patterns. All the flakes with different thicknesses show a two-fold polarization dependence pattern due to the unequal THG emission for the laser polarization along the armchair and zigzag directions. An average THG anisotropy ratio of $1.93\pm 0.44$ is observed among the measurements for different thicknesses where the anisotropy ratio is quantized as the ratio of the maximum THG intensity to the second maximum one as ${\left|{I}^{3\omega }\right|}_{0^\circ }/{\left|{I}^{3\omega }\right|}_{90^\circ }$. The relatively high variation in THG anisotropic ratio can be addressed to the defects or strain which may be introduced during the mechanical exfoliation process. Such effects on the nonlinear optical properties has been previously observed for different nonlinear optical materials^13,16,39,40^. According to Eqs. (9) and (10), the third-order nonlinear susceptibility components also show anisotropy with the average ${\chi }_{11}/{\chi }_{22}$ ratio of $1.38\pm 0.16$. Furthermore, the THG conversion efficiency of SiP flakes with different thicknesses are measured for an average pump power of $3.6 \mathrm{mW}$, corresponding to an excitation irradiance of $28.2\mathrm{GW}/{\mathrm{cm}}^{2}$. The conversion efficiency for the SiP flake with 12 nm thickness is $1.02 \times {10}^{-10}$ which is the minimum conversion efficiency, while the conversion efficiency for the thickest flake with 90 nm thickness is measured as $6.56 \times {10}^{-10}$. On the other hand, the maximum conversion efficiency is achieved for the 33 nm-thick SiP flake as $1.82 \times {10}^{-9}$ which is comparable to the reported THG conversion efficiencies for other kinds of anisotropic 2D materials including GeAs ($1.1 \times {10}^{-9}$ for an irradiance of $8.4\mathrm{GW}/{\mathrm{cm}}^{2}$)^15^, GeSe ($2.7 \times {10}^{-9}$ for an irradiance of $5.6\mathrm{GW}/{\mathrm{cm}}^{2}$)^41^, BP ($2.8 \times {10}^{-9}$ for an irradiance of $440\mathrm{GW}/{\mathrm{cm}}^{2}$)^40^ and ReS_2_ ($0.2 \times {10}^{-9}$ for a irradiance of $130\mathrm{GW}/{\mathrm{cm}}^{2}$)^16^, as well as graphene ($3 \times {10}^{-9}$ for an irradiance of $186\mathrm{GW}/{\mathrm{cm}}^{2}$)^6^.

**Figure 4:**
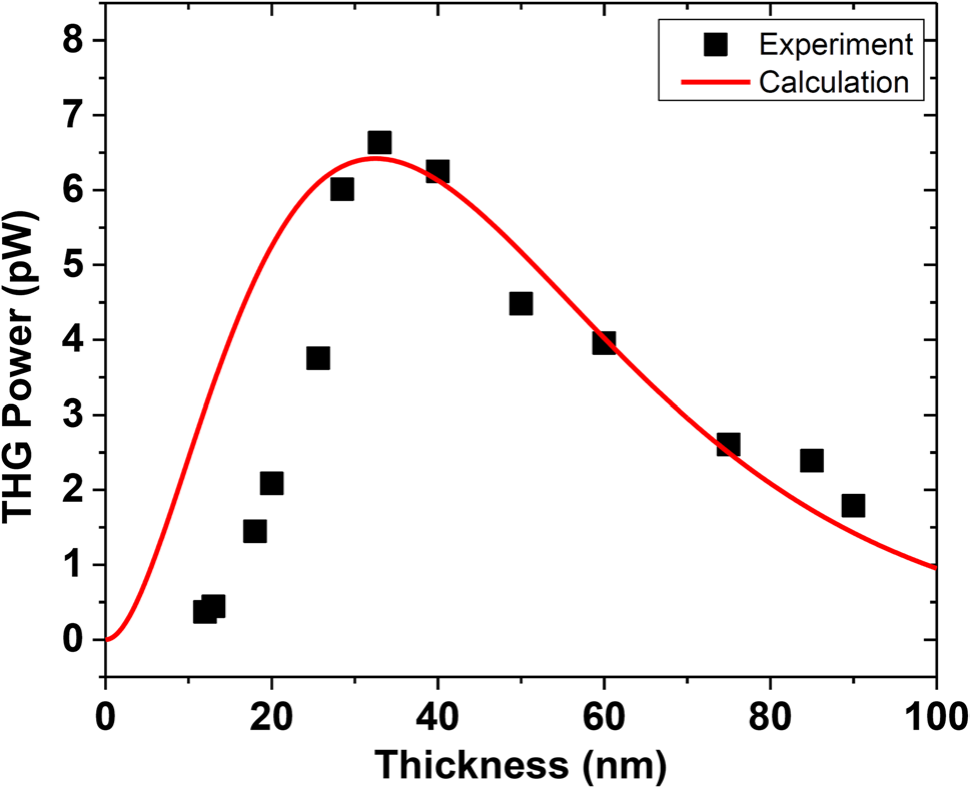
The dependence of THG emission power on the thickness of exfoliated SiP flake with the data fitting by Eq. (12). The black squares and solid red line refer to the experimental data and theoretical fitting, respectively.

Figure 4 plots the THG emission power as a function of the thickness of exfoliated SiP flake. The thickness dependence trend can be explained by two mechanisms. The first mechanism is the rapid increase of THG power in the thickness range of $12$ to $33 \mathrm{nm}$ which is a result of the cumulative contribution of the increased SiP layer number. However, the second mechanism is the exponential depletion of THG emission as the thickness increases from $33$ to $90 \mathrm{nm}$, which is related to the increased optical propagation length through the thick SiP flake that results in the domination of strong optical absorption. To further intuitively understand the effect of the flake thickness on the THG power and estimate the extinction coefficient, the THG power with respect to the flake thickness can be derived by solving the nonlinear Maxwell’s equations as^13^:

11 $${P}^{3\omega }\left(d\right)=\frac{9{\omega }^{2}{d}^{2}}{16\sqrt{{n}_{3}^{2}+{k}^{2}}{n}_{1}^{3}{\epsilon }_{0}^{2}{c}^{4}}{\left|{\chi }^{\left(3\right)}\right|}^{2}\frac{{({P}^{\omega })}^{3}}{{f}^{2}{W}^{4}{\tau }^{2}{\left[\frac{\pi }{4\mathrm{ln}2}\right]}^{3}}\left(\frac{{e}^{-\frac{4\pi kd}{{\lambda }_{3}}}-2\mathrm{cos}\left(\Delta kd\right){e}^{-\frac{2\pi kd}{{\lambda }_{3}}}+1}{{d}^{2}\left(\frac{4{\pi }^{2}{k}^{2}}{{\lambda }_{3}^{2}}+{\Delta k}^{2}\right)}\right){e}^{-\frac{4\pi kd}{{\lambda }_{3}}}$$

where $d$ is the flake thickness, $k$ is the extinction coefficient which is the imaginary part of refractive index at $3\omega$,${n}_{1}$ and ${n}_{3}$ are the real parts of refractive indices at the fundamental and THG wavelengths, ${P}^{\omega }$ is the average pump power, $\Delta k=\frac{6\pi }{\lambda }\left({n}_{1}-{n}_{3}\right)$ is the phase mismatch between the forward propagating fundamental and the third-harmonic signal, and the pulsed pump laser parameters include beam width $W$, pulse width $\tau$, and repetition rate $f$. With the constant average pump power ${P}^{\omega }$, Eq. (11) can be further simplified as:

12 $${P}^{3\omega }\left(d\right)\approx A{d}^{2}\mathrm{exp}\left(-\frac{4\pi kd}{{\lambda }_{3}}\right)$$

where $A$ is a constant. The measured THG power is fitted by Eq. (12) as the solid red line in Fig. 4, which gives the estimated extinction coefficient $k=2.55$ at the THG wavelength of $520 nm$ for the exfoliated SiP thin flakes*.* It is observed that for SiP flakes having small thicknesses less than 30 nm, the measured THG power shows the same trend as the fitted data, but the measured values are lower than the fitted ones. One potential explanation for this discrepancy is that the mechanical exfoliation and transfer process of SiP thin flakes from tape to glass substrate may affect several top surface layers negatively, which represents a large portion of the entire flake, so that the THG conversion efficiency is notably reduced for thin flakes less than 30 nm with weak THG responses. Another potential reason is the possible oxidation of the first few surface layers in SiP thin flakes exposed to ambient air, which also degrades the THG conversion efficiency. Future investigations using clean exfoliation in inert environment and surface passivation to effectively protect the SiP surface layers against the possible defects and ambient oxidation, and thus preserve their pristine structural and optical characteristics could potentially solve such discrepancy issue.

### Discussion

In summary, the strong anisotropic nonlinear optical properties of the mechanically exfoliated 2D SiP flakes with different thicknesses are investigated in terms of the THG process. The angle-resolved polarized Raman spectroscopy is used to confirm the Raman signature of 2D SiP, determine the crystal orientation, and define the armchair and zigzag edge configurations of 2D SiP by using anisotropic Raman mode analysis. The angle-resolved THG emission with respect to the incident pump polarization angle is found to be strongly anisotropic with the two-fold polarization dependence pattern. The consistency of the anisotropic THG emission from SiP flakes with different thicknesses is revealed by the angle-resolved THG emission analysis. Furthermore, the THG conversion efficiency from the exfoliated SiP flakes with different thicknesses is also measured. The effect of the SiP flake thickness on the THG emission power is analyzed by the derived expression, where the extinction coefficient of SiP thin flakes at the THG wavelength is estimated and the optimal thickness for high conversion efficiency is revealed. The reported anisotropic nonlinear optical properties of 2D layered SiP shows its great potential as a nonlinear optical material used for future photonic integrated circuits and quantum chips.

## Methods

### Sample preparation

The bulk SiP single crystal (2D Semiconductors) is mechanically exfoliated to isolate SiP thin flakes on the pre-cleaned glass cover slip substrate by the Nitto tape (SPV 224). The glass cover slip substrate is cleaned with the aid of sonication in acetone, isopropanol alcohol and deionized water.

### Angle-resolved Raman spectroscopy measurements

The total Raman spectrum and the angle-resolved polarized Raman spectra are measured by a home-built optical setup with a $632.8 \mathrm{nm}$ He–Ne laser. The laser beam passes through a linear polarizer and a half-wave plate to set the linear polarization before the sample. The laser beam is then focused on the sample by a 60 × objective lens ($NA = 0.85$). A linear polarization analyzer is used after the sample to select the parallel polarization component of the Raman spectrum. The back-reflected signal is collected by the same objective lens and sent to the spectrometer. The excitation laser is blocked by a Rayleigh band rejection filter (Semrock, LP02-633RE-25) before the spectrometer.

### Angle-resolved THG measurements

The excitation source for THG measurements is chosen as a femtosecond pulsed laser at $1560 \mathrm{nm}$ with a spot size of $2.5 \mathrm{\mu m}$ (Calmer fiber laser, pulse width $90 fs$, repetition rate $80 \mathrm{MHz}$). The input laser polarization is manipulated by a combination of a linear polarizer and a half-wave plate before the sample, while the output THG emission is passed through an analyzer after the sample. The pulsed laser beam is focused on the sample using a 40 × objective lens ($NA = 0.65$) and the emitted THG signal is collected using a 100 × objective ($NA = 0.70$). The excitation pulse transmitted through the sample is blocked by a low pass filter and the signal is collected into a spectrometer (Horiba, iHR 520) for the measurements.
